# GM plants with RNAi - golden mosaic resistant bean

**DOI:** 10.1186/1753-6561-8-S4-O24

**Published:** 2014-10-01

**Authors:** Francisco José Lima Aragão

**Affiliations:** 1 Embrapa Recursos Geneticos e Biotecnologia, Brasilia, DF, Brazil

## 

RNA silencing is a biochemical mechanism that regulates gene expression by post-transcriptionally activating a sequence-specific RNA degradation by three different pathways: (i) small interfering RNA (siRNA) silencing of exogenous mRNA; (ii) micro RNA (miRNA) silencing of endogenous mRNAs, and (iii) associated with DNA methylation and suppression of transcription [2].

These processes share three basic biochemical phases: (i) formation of double-stranded (ds)RNA; (ii) processing of dsRNA into small dsRNA molecules (sRNA); and (iii) targeting of single-stranded small RNA to sequence-specific DNA or RNA molecules. Although several mechanisms can generate dsRNA, the sRNA processing and effector phases have a common biochemical core.

One key-component of this system is small RNA molecules of 21 to 26 nt called siRNAs (small interfering RNAs) which originated from longer double-stranded RNA (dsRNAs) cleaved by a specific RNase endonuclease called DICER with distinctive dsRNA binding, RNA helicase, RNase III and PAZ (Piwi/Argonaute/Zwille) domains. The siRNA strands are further relaxed (unwound) and one strand is incorporated in a RISC complex (RNA-induced silencing complex) which contains a member of the Argonaute (Ago) protein family, guiding this complex to an mRNA with a complementary sequence, which is then cleaved, leading to gene silencing.

RNAi can be triggered by double-stranded or partially self-complementary hairpin RNA formation. In addition to playing a powerful role in creating loss of function mutations in plants, RNAi biological functions include also the regulation of endogenous gene expressions, such as micro RNA (miRNA), heterochromatin formation, transposon repression and defence against viral infection [2,3].

Several strategies have been employed for genetically engineering resistance to viruses in transgenic plants, including the expression of coat protein genes, the expression of truncated defective genes and antisense RNA. Now that we better understand the mechanisms of RNA silencing (RNAi) and its biological functions, it is possible to look back on initial experiments from a new perspective. It is now known that plants naturally process viral RNAs to generate small sequences of a pathogen's genetic material that can be specifically used against that pathogen through the RNA-induced silencing complex. It was recognized that an RNAsilencing (post-transcriptional gene silencing or PTGS) mechanism was responsible for the resistance against RNA viruses, and that it depends on the formation of double-strand RNA (dsRNA) whose antisense strand is complementary to the transcript of a targeted gene. This discovery led to the introduction in transgenic plants of constructs to produce intracellular generation of siRNA-like species to induce targeted gene silencing and virus resistance.

RNA silencing has been an important tool to generate plants resistant to a large range of viruses. RNA sense- or antisense-mediated strategies resulted in a maximum resistance frequency of 20%, but often far lower frequencies were obtained. In addition, not all viral genes used in transgenic constructs rendered resistant plants. The use of inverted repeat constructs, resulting in dsRNA transcripts, rendered a very efficient system in which much higher frequency of transformed lines will display efficient gene knockdown or virus resistance. The probable reason is that the dsRNAs fed directly into the silencing pathway at the level of the RNase III-like enzyme Dicer, and therefore there is no reliance on the action of plant-encoded RNA-dependent RNA polymerase proteins. So far, most examples of RNAi-mediated virus resistance are related to RNA plant viruses.

Golden mosaic, caused by the *Bean golden mosaic virus *(BGMV), transmitted by the whitefly *Bemisia tabaci *Gen., is one of the most limiting diseases for bean cultivation in the American continent. Since highly resistant bean varieties are unavailable, control practices have focused on controlling the vector with high-toxicity insecticides, with cost-benefit ratio and environmental concerns. In Brazil, the golden mosaic causes annual losses in bean production ranging from 90,000 to 280,000 tons. In addition, there are approximately 180,000 hectares unsuitable for bean cropping in the dry season due to the high prevalence of BGMV

The concept of using RNAi constructs was explored to generate genetically modified (GM) bean lines resistant to the BGMV by silencing the *rep *viral gene, which encodes the only protein strictly essential for viral genome replication [4]. Two lines (named 2.3 and 5.1) showed immunity to the BGMV upon inoculation at high pressure (>300 viruliferous whiteflies per plant during its entire life cycle). Under field conditions, no virus infection has been observed in transgenic genotypes from 2007 to 2013, even under high *B. tabaci *pressure (Aragão and Faria, 2009; personal communication). In contrast, non-transgenic varieties have shown up to 100% infection, presenting characteristic severe symptoms of the golden mosaic disease.

In 2011, a transgenic bean event (named EMB-PV051-1; Embrapa 5.1) resistant to the BGMV was approved for cultivation and consumption in Brazil. Biosafety evaluations were carried out taking into account the protocol proposed by the Brazilian Biosafety Committee (CTNBio) to demonstrate the safety of transgenic bean event to the environment and human health. Analyses have suggested no differences between the transgenic event and its non-transgenic counterpart.
